# Combining Business Methods with Professional Methods in Dealing with Our Patients

**Published:** 1892-12

**Authors:** C. B. Blackmarr


### Combining Business Methods with Professional Methods in Dealing with Our Patients.

BY C. B. BLACKMARR, D.D.S.

Read before the Michigan State Dental Society, June, 1892.

I shall not be disappointed if the older practitioners of dentistry are not interested in my paper, because they may be supposed of course to have mastered the problem of how to manage their patients in a business, as well as in a professional way. They have their certain families for whom they perform the operations necessary for the keeping of their teeth in proper condition. These patients are told to present themselves from one to four times a year as the individual case may require. Time is saved and appointments are made ahead to correspond with advice given the patients. A dentist thus caring for a certain number of families, has his time so taken that he has no time to attend to transient patients or those who happen into his office wanting a "nerve killed" or a "tooth plugged" at once. His lady assistant will save this dentist's time by not having him see the patient at all, and will answer the patient by saying that the doctor's time is already taken by previous appointments for the day. As a dentist can work for only one patient at a time the answer is very conclusive. If, however, this transient who has happened in *happens* to represent a family which the dentist is anxious to secure as one of *his*, it *changes* matters some. Short appointments between others will be made at once and longer ones in the future, and so this dentist keeps himself busy with those he desires most to have; keeping those who appreciate his work the most, and those who are willing to pay him what his time and skill are worth. Keeping those who care most to keep their teeth, and who appreciate and desire the better class of dental operations. Letting go those who do not. A dentist having such a well-managed practice has reason to be satisfied as far as business success in life is concerned and with the pleasantness and satisfaction of his work. This man will be satisfied with his income and work to the end, barring accidents, sickness, etc., if he keeps up with the times.

There are, however, some of the older practitioners who are not as satisfied as I have pictured this dentist. And why? Is it on account of lack of professional knowledge or methods? No. It is rather a lack of business knowledge and method. To illustrate: I have one of these dentists in mind now, a very conscientious and excellent dentist, one who practiced in a good-sized city for twenty-five years or more, and at one time, for several years, had an excellent practice to manage. But he "never saved for a rainy day." He never has owned a home—unless it was one on which there was a mortgage, which always took the home. He has his life insured I believe, by the way. And now it is pitiful to see him try to make income pay expenses, and as he is growing older the struggle is growing harder. He often will look at a tooth with mirror and explorer and talk an hour about it to the patient telling about the fee, what would have to be done if he should find the nerve exposed, etc., when the same length of time would have filled it. Well, at last he makes an appointment and says, "come at half past one or two" and as he was not exact in regard to the time the patient was not in coming, and came at nearly half past two. The tooth was filled and finished about half past four—too late for him to have another patient, so this constituted his afternoon's work. Upon the patient asking the amount of his fee, he said to himself: "one hour's consultation and two hours operating, I ought to have six dollars," and so he said to his patient. Now, he wonders why the patient don't come back to him again, and he wonders why he don't make more money, because six dollars was a good big fee for such a filling, while the facts in the case were these: She had happened to have a filling of like size, etc., put in for three dollars, in one hour, by another dentist who works by the hour and makes his fee according to the demand for his time and skill. He put this filling in from one to two o'clock, and three others from two to five o'clock, making his afternoon's time bring him in an income of twelve instead of six dollars. The first is called unreasonable in his charges, the other reasonable, and yet the latter gets more pay for his time.

And another case, I remember, was one of the best "gold fillers" I ever knew, but his management was bad. He kept a lady patient once in his chair from nine A.M. till six P.M., putting in two fillings. This was nothing uncommon for him. The dentist had his dinner, the patient none. Now, those fillings were elegant professionally but failures financially, and he wondered why she never could bear to come back to his office again when he had worked so long and hard to please her. And her brother refused to rent to him an office in his block, because he was afraid he would not have patients enough so he could pay rent, and at present he cannot meet expenses, rent, etc., and yet his professional skill is unquestionable. I think we waste a good deal of time by simply not doing what we have to do by combining business methods with professional methods. I have seen a dentist argue with a patient over the difference in the fee for filling with gold or cement, when both could have been done in the time thus spent. I have seen a dentist sit around waiting for a cement filling to harden long enough to fill and finish a gold filling in the same cavity, and the only reason for not putting in gold was the extra expense to the patient.

I said at the beginning that I did not expect to interest the older practitioners, but to the younger ones who have just graduated this subject may be more interesting, because at college they have been taught to fill teeth, etc , to please their teachers in a professional way, but now that they are in their own offices they have their patients to please instead, and that is quite a different affair as far as business matters are concerned, and these matters are quite important usually as one commences his practice. He must please his patients. He must study each individual case and find out what will please, and make his fees correspond with his skill and with the worth of his time, charging more as they are worth more, or less as the case may be. Then he can regulate his practice into a specialty or in any way he chooses later. This ability to please will give a young graduate a good start by giving him at first plenty of patients, who can be managed in future as best suits him. And he should start in to be honest in all his doings. Patients like to trust themselves to an honest professional man; to illustrate: I knew a young graduate who had had his office opened for a month or so, and a bookkeeper thought he would try the new dentist and went to make an appointment. The dentist told him he could not give an appointment before six weeks, as his time was all taken with previously-made appointments. The bookkeeper, doubting the dentist's statement, watched for and got an opportunity to glance at the appointment book and saw no names between the present week and the sixth. This dentist wondered why the bookkeeper cancelled his appointment. Again, I knew a young man who was trying to establish himself into a high-class practice by making high fees, and he gave his patients the reason for his high charges that he used a very superior quality of gold, compared with what other dentists used in the city, and this dentist wonders why some of his patients don't come back to him, after they have investigated the subject of prices of gold foil and the amount required to fill ordinary cavities.

A dentist gets a suit of clothes and has the price charged to him with the hope the clothing merchant will "patronize him." The merchant after sending bill, followed by collector, without getting his pay, concludes he will have to "take it out in dentistry," and so sends in some of his family and "gets even" at last, and the dentist wonders why he don't come to him again, and why? Because this dentist did not treat this business man in a business like manner, although he may have treated his teeth very professionally. I once saw a very conscientious dentist work and struggle with a nervous, kicking, crying, eleven year old boy for three hours and finally got in two gold fillings. These fillings were polished in a very professional like manner and the dentist received four dollars as his fee. He expected the boy to see for himself in a short time what an elegant piece of work he had done for him, and expected him to come back to him again. But instead the boy went to another dentist who put in two cement fillings in the opposite places to the gold.

This took him thirty minutes and he charged the boy two dollars, and told him to come back once in a while and let him see them, and after he was older and the teeth less sensitive he would put in gold and hurt him very little. Now which, think you, got the patient in the future and which got the most for his time? Which was the most satisfied with the results of his work? Which was the patient most satisfied with?

I have seen a dentist talk with his patient or hunt in his records for thirty minutes, to find out the condition of a tooth, when one minutes examination of the tooth would have told him better.

I have heard it said that if a dentist could have one of his teeth filled once a week he would treat his patients better. And I have often wished that some of these dentists who do not economize their time would go into any good business like office, whether it be a mercantile, express, post, law or dental office, and notice how quickly and decidedly their questions will be disposed of, and themselves too by the ones having charge of the office. It does seem as if the prompt business like answers would make such a dentist wonder why he could not dispose of his own business and professional matters in the same way. At least set him to thinking about it, and that is simply what I have tried to do by writing this.

I have sometimes thought that writers and talkers on many subjects, whether in a prayer meeting or at a dental meeting, were practically the poorest ones to *work* it out, and now as I think of my paper and myself I can see no reason for changing my theory.

